# Supplementary material for: Single-cell T-cell receptor repertoire profiling in dogs
Source: Commun Biol. 2024 Apr 22;7:484. doi: 10.1038/s42003-024-06174-w (PMC11035579; doi:10.1038/s42003-024-06174-w)
Supplement: Supplementary file 2 — Supplementary Information [file 42003_2024_6174_MOESM2_ESM.pdf]

Single-cell T-cell receptor repertoire profiling in dogs

My H. Hoang[1,2,†], Zachary L. Skidmore[1,2,†], Hans Rindt[3,†], Shirley Chu[1,3], Bryan Fisk[1,2], Jennifer A. Foltz[1], Catrina Fronick[2], Robert Fulton[2], Mingyi Zhou[4], Nathan J. Bivens[4], Carol N. Reinero[3], Todd A. Fehniger[1,5], Malachi Griffith[1,2,5,6], Jeffrey N. Bryan[3,*], Obi L. Griffith[1,2,5,6,*]
[†] These authors contributed equally
[*] corresponding authors

Affiliations
1. Division of Oncology, Department of Medicine, Washington University School of Medicine, St Louis, MO, USA.
2. McDonnell Genome Institute, Washington University School of Medicine, St Louis, MO, USA.
3. Department of Veterinary Medicine and Surgery, University of Missouri, Columbia, MO, USA.
4. Genomics Technology Core, University of Missouri, Columbia, MO, USA.
5. Siteman Cancer Center, Washington University School of Medicine, St Louis, MO, USA.
6. Department of Genetics, Washington University School of Medicine, St Louis, MO, USA.

Address correspondence to: Jeffrey N. Bryan, bryanjn@missouri.edu; Obi L. Griffith, obigriffith@wustl.edu

Supplementary Tables, Figures and Data

Suppl Table 1. Dog TCR amplification primer sequences

Target	Reaction	Primer Name	Primer Sequence (5’ to 3’)
TCR α chain (TRA)	First reaction (Outer)	TRA Forward 1 (10x 5’ v1)	AATGATACGGCGACCACCGA-GATCTACACTCTTTCCCTACACGACGCTC
		TRA Forward 1 (10x 5’ v2)	GATCTACACTCTTTCCCTACACGACGC
		TRA Reverse 1	TCGGTGAACAGGCAGACAGTCC
	Second reaction (Inner)	TRA Forward 2 (10x 5’ v1)	AATGATACGGCGACCACCGA-GATCT
		TRA Forward 2 (10x 5’ v2)	GATCTACACTCTTTCCCTACACGACGC
		TRA Reverse 2	TGGTACACAGAGGGGTCAGG
TCR β chain (TRB)	First reaction (Outer)	TRB Forward 1 (10x 5’ v1)	AATGATACGGCGACCACCGA-GATCTACACTCTTTCCCTACACGACGCTC
		TRB Forward 1 (10x 5’ v2)	GATCTACACTCTTTCCCTACACGACGC
		TRB Reverse 1	TTCTGGGTCCGCGAGATCTC
	Second reaction (Inner)	TRB Forward 2 (10x 5’ v1)	AATGATACGGCGACCACCGA-GATCT
		TRB Forward 2 (10x 5’ v2)	GATCTACACTCTTTCCCTACACGACGC
		TRB Reverse 2	GGTTCAAACACTGTGACCGT

Note: *TRA/TRB Forward 1/2 primer sequences are identical and as provided by the 10x Chromium Single Cell V(D)J Reagent Kits User Guide (CG000086 Rev L) or 10x Chromium Next GEM Single Cell 5' Reagent Kits v2 (Dual Index) User Guide (CG000331 Rev C) for v1 and v2 kits respectively.

Suppl Table 2. Detailed clinical information for normal canine samples

ID	Normal_A	Normal_B	Normal_C [a]	Normal_D	Normal_E	Reference Ranges [b]
Age (yrs)	3	4	1	4	8	N/A
Sex	FS	MC	MC	FS	MC	N/A
Breed	Australian Shepherd	Mixed breed	Great Pyrenees	Dachshund	Great Dane	N/A
CBC (/ul)						
WBC	7630	6310	Within normal range	8100	7740	4800 - 13400
Lymphocytes	2820	1830	35%	1130	1470	1100 - 4750 (8-99%)
Seg. neutrophils	4350	3790	45%	6320	4880	2490 - 9280 (18-100%)
Monocytes	300	380	7%	320	930	90 - 990 (0.7-21%)
Lymph node cytology	No overt cytologic abnormalities	No overt cytologic abnormalities	No overt cytologic abnormalities	Reactive lymphoid hyperplasia	Reactive lymphoid hyperplasia	N/A
Lymph node flow cytometry (%)						
CD5+	72.5	60.9	67.6	67.9	23.9 ↓	52.3 +/-12.7 [1]<br>35-74 [c]
(CD5+)CD4+	70.3 ↑	67.4	62.7	62.9	74 ↑	58.7 +/- 9 [1]<br>21-45 [c]
(CD5+)CD8+	22.9	22.1	25.1	30.5 ↑	14.7	21.3 +/- 7.8 [1]<br>6-20 [c]
CD21+	29.3	41.2	29.2	27.9	65 ↑	33.9 +/- 11.8 [1]<br>26-58 [c]
(CD4+)CD25+	6.15	7.18	8.84	6.78	3.23	1.98 [2]<br>6.6 [3]

(CD8+)CD25+	0.46	0.69	0.42	0.56	0.23	N/A
(CD4+)CD25+ FoxP3+	5.2	6.83	7.9	6.54	6.87	1.97 [2]
(CD4+CD25+) FoxP3+	51.1	54.4	54.9	50.1	46	47.01 [2]
(CD4+)FoxP3+	3.67	4.09	4.45	3.7	2.26	2.63 [2]
PBMC flow cytometry (%)						
CD5+	68.1 ↓	60.5 ↓	67.3 ↓	68.8 ↓	64.3 ↓	77.9-92.4 (mean 83.3) [2]
(CD5+)CD4+	59.4	65.4	63.4	64.3	59.1	26-68.4 (mean 54.6; median 55.3) [4]<br><br>31.5-66.0 (mean 45) [5]
(CD5+)CD8+	25.2	16.5 ↓	22.5	22.2	28.2	20.5-43.6 (mean 28.8) [5]
CD21+	19.3	28 ↑	9.2	20	10.1	5.6-21.3 (mean 12.9) [5]
(CD4+)CD25+	3.54	5.53	3.7	3.65	6.66	7.19-28.1 (mean 15.0; median 15.1) [4]<br><br>3-7% [3]
(CD8+)CD25+	0.75	0.93	0.55	0.6	1.67	N/A
(CD4+)CD25+ FoxP3+	1.19	2.13	1.61	1.09	0.84 ↓	0.91-3.86 (mean 2.44; median 2.50) [4]
(CD4+CD25+) FoxP3+	24.8 ↓	24.4 ↓	27.2 ↓	22.8 ↓	11.2 ↓	71.68 [2]<br><br>52.9% [3]
(CD4+)FoxP3+	1.59 ↓	2.66	1.43 ↓	2.55	1.68 ↓	1.71-6.42 (mean 4.46; median 4.98) [4]

Note: FS = female spayed; MC = male castrated; WBC = white blood cells.[a] Hematology analyzer results were not available but results from a differential performed by a clinical pathologist are provided. [b] Reference ranges vary substantially depending on the instrument used, freeze/thaw cycles, antibodies used and gating strategy. The reference ranges for the CBCs were from the MU diagnostic lab. Reference ranges for

the other parameters are specified. [c]Colorado State University Clinical Hematopathology Laboratory. [1]Rutgen *et al* 2015. Mean values are presented.
[2]Rissetto *et al.* 2010. Median values are presented.
[3]Pinheiro *et al.* 2011.
[4]Sparger *et al.* 2021. CD3 was used instead of CD5 to identify T cells.
[5]Byrne *et al*. 2000. CD3 was used instead of CD5 to identify T cells.

Suppl Table 3. Detailed clinical information for canine lymphoma samples

**ID**	**Tzone_LSA**	**PTCL_NOS**
Age (yrs)	7	4
Sex	FS	MC
Breed	Golden Retriever	Golden Retriever
Diagnosis	T zone lymphoma	PTCL-NOS (suspected)
Histopathology	The cortical follicles are multifocally compressing and displaced by expanded sheets of intermediate sized lymphocytes arranged in sheets supported by a scant fibrous stroma. The cells have scant pale eosinophilic cytoplasm with indistinct cell margins and have a round finely stippled nucleus with typically indistinct nucleolus. Cellular and nuclear pleomorphism are mild. Mitotic figures number 8 in ten HPFs.	ND
Flow cytometry	CD8+CD5+CD45- lymphocytosis. Cells were intermediate/large in size	ND
Staging	Thoracic radiographs: WNL	Thoracic radiographs: widened mediastinum<br>Abdominal radiographs: WNL
Stage	3a (incompletely staged)	3b (incompletely staged)
Treatment	Careful monitoring followed by metronomic chlorambucil and prednisone started 10 months post diagnosis	CHOP chemotherapy
Concurrent Disease(s)	*Ehrlichia canis* (exposure or previous infection). Seasonal dermatitis	Paraneoplastic hypercalcemia
Survival (days)	>476	>49

Note: WNL = within normal limits; ND = not done; PTCL-NOS = peripheral T cell lymphoma - not otherwise specified.

Suppl Table 4. Detailed clinical information for canine melanoma samples

ID	Melanoma_A	Melanoma_B	Melanoma_C	Melanoma_D
Age	7	14	12	13
Sex	Female spayed	Male castrated	Male Castrated	Female spayed
Breed	Bouvier des Flandres	American Cocker Spaniel	Dachshund	Mixed breed dog
Diagnosis	Melanoma of unknown primary (superficial cervical lymph node)	Buccal mucosal melanoma	Gingival (maxillary) mucosal melanoma	Gingival (mandibular) mucosal melanoma
Stage (at diagnosis)	Stage 3 at diagnosis.	Stage 2 at diagnosis. Stage 3 at recurrence and start of the deglycosylated autologous vaccine trial.	Stage 2 at diagnosis and start of the 1st deglycosylated autologous vaccine trial.<br>Stage 2 at recurrence and start of the 2nd deglycosylated autologous vaccine trial. Stage 3 at recurrence and start of radiation therapy.	Stage 2 at diagnosis.<br>Stage 3 at the start of the deglycosylated autologous vaccine trial
Treatment	Lymphadenectomy followed by deglycosylated autologous vaccine. Marginal excision of cutaneous melanoma in ipsilateral antebrachium followed by deglycosylated autologous vaccine (320 days post original diagnosis). Lymphadenectomy with lip melanoma marginal excision followed by Oncept vaccine (1698 days post original diagnosis).	Marginal excision of primary lesion. Marginal excision of recurrent now amelanotic melanoma with lymphadenectomy followed by deglycosylated autologous vaccine (95 days post original diagnosis). Marginal excision of recurrent amelanotic melanoma followed by deglycosylated autologous vaccine (598 days post original diagnosis).	Marginal excision of the primary lesion. Marginal excision of recurrent lesion and regional lymphadenectomy. Marginal excision of recurrent lesion, followed by hypofractionated radiation therapy (8 Gy x 3).	Rostral bilateral mandibulectomy. RLN metastasis (412 days post original diagnosis). Lymphadenectomy followed by deglycosylated autologous vaccine.
Concurrent	Bilateral glaucoma	Protein losing	Intervertebral	Grade III/VI systolic

Disease(s)		nephropathy, sebaceous adenomas	degenerative disc disease. Severe periodontal disease	murmur. Suspected protein losing nephropathy.
Cause of Death (from the date of diagnosis)	Metastatic melanoma (multifocal skin, heart, kidney, lung, liver, inguinal lymph node)	Hemoabdomen (unknown cause). Pulmonary metastasis and urogenital transitional cell carcinoma on necropsy but determined to not be the cause of death.	Central or peripheral vestibular signs (unknown cause). No evidence of melanoma on physical exam. No necropsy	Suspected right forebrain lesion (unknown cause). No evidence of melanoma or other neoplasia on necropsy; gliosis of the cerebrum with lipofuscinosis; moderate glomerulopathy with medullary fibrosis
Survival (days)	1858	741	1526	1205

Suppl Table 5. Antibodies used in flow cytometry panels.

Target/species	Conjugates	Clone #	Vendor
CD5/canine	perCP-eFluor710	YKIX322.3	BioRad
CD21/canine	PE	CA2.1D6	BioRad
CD4/canine	FITC	YKIX302.9	BioRad
CD8/canine	APC	YCATE55.9	BioRad
CD25/canine	PE	P4A10	eBioscience
FoxP3/multiple	APC	FJK-16S	eBioscience

Suppl Table 6. Single Cell RNA-sequencing cell typing results from PBMCs of 4 dogs with melanoma and 1 lymph node aspirate from a dog with T zone lymphoma

		Cell counts (%)				
**Cell Type**	**Cell Abbrev.**	**Melanoma_A**	**Melanoma_B**	**Melanoma_C**	**Melanoma_D**	**Tzone_LSA**
B-Cell	B	1306 (20.97)	693 (10.41)	999 (12.17)	385 (4.56)	322 (4.20)
Basophil	BASO	10 (0.16)	1 (0.02)	9 (0.11)	5 (0.06)	1 (0.01)
CD4+ T-cell	CD4	1639 (26.32)	1284 (19.29)	1792 (21.84)	2248 (26.61)	182 (2.38)
CD8+ T-cell	CD8	1358 (21.80)	2086 (31.34)	1995 (24.31)	2842 (33.65)	7048 (92.01)
Common Myeloid Progenitor	CMP	1 (0.02)	0 (0.00)	0 (0.00)	0 (0.00)	1 (0.01)
Dendritic cell	DC	63 (1.01)	3 (0.05)	165 (2.01)	266 (3.15)	12 (0.16)
Eosinophil	EOS	3 (0.05)	0 (0.00)	1 (0.01)	0 (0.00)	0 (0.00)
Erythroid	ERY	217 (3.48)	80 (1.20)	35 (0.43)	361 (4.27)	0 (0.00)
Granulocyte	GRAN	10 (0.16)	36 (0.54)	13 (0.16)	46 (0.54)	4 (0.05)
Hematopoietic Stem Cell	HSC	2 (0.03)	0 (0.00)	0 (0.00)	0 (0.00)	0 (0.00)
Megakaryocyte	MEGA	14 (0.22)	28 (0.42)	113 (1.38)	57 (0.67)	0 (0.00)
Megakaryocyte/Erythroid Progenitor	MEP	2 (0.03)	0 (0.00)	0 (0.00)	1 (0.01)	0 (0.00)
Monocyte	MONO	1468	2318	2918	2066	35 (0.46)

		(23.57)	(34.83)	(35.56)	(24.46)	
Not Available/ Not Determined	NA	73 (1.17)	38 (0.57)	63 (0.77)	124 (1.47)	46 (0.60)
Natural Killer	NK	18 (0.29)	78 (1.17)	63 (0.77)	29 (0.34)	4 (0.05)
Precursor B-Cell	PreB	44 (0.71)	10 (0.15)	40 (0.49)	17 (0.20)	5 (0.07)
Totals (filtered)	16 cell types	6228	6655	8206	8447	7660

Suppl Figure 1. TCR α chain enrichment strategy for use with 10x scRNA sequencing.

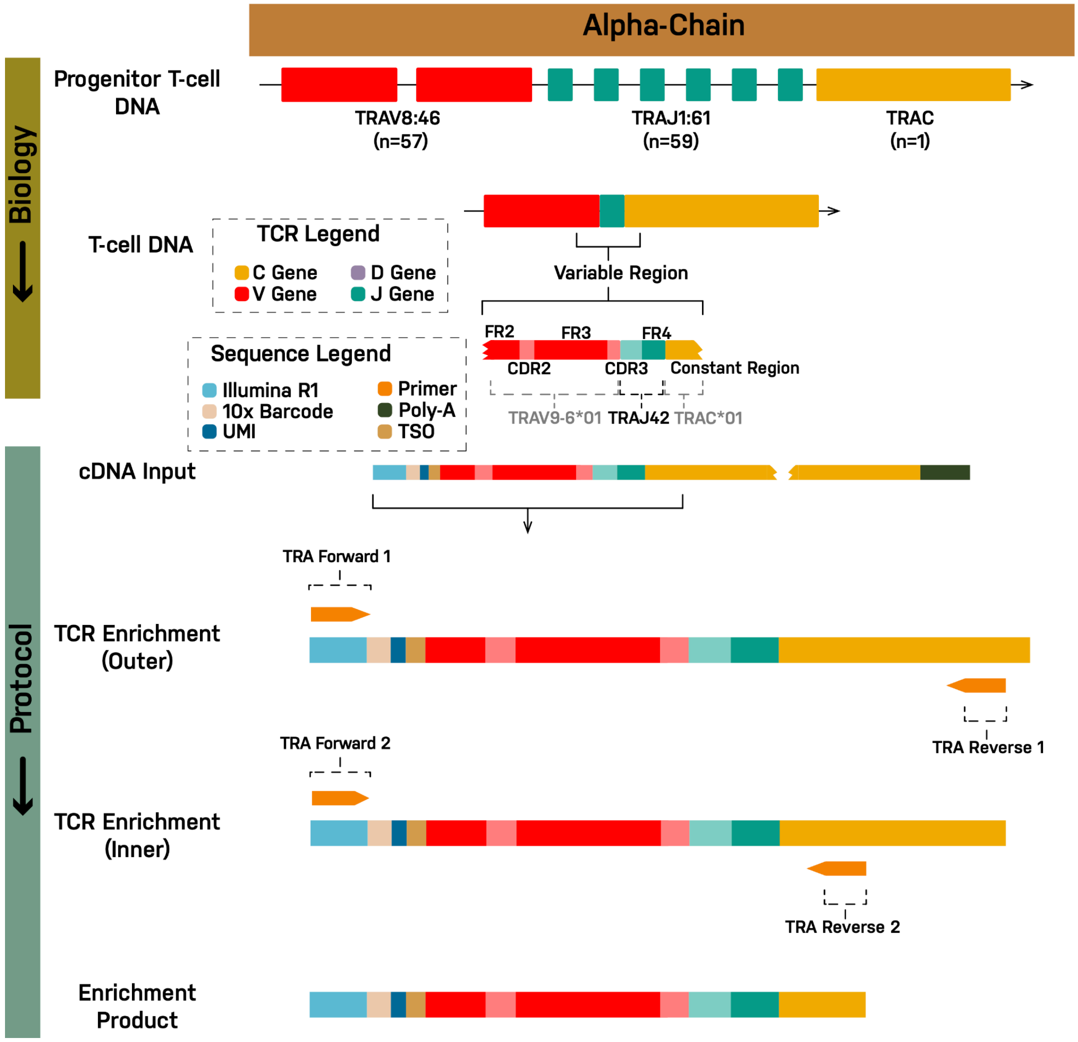

The TCR V(D)J enrichment strategy is depicted for the α chain (See **Figure 1** for β chain). At top, the genomic un-rearranged TRA locus is shown. During T cell development from progenitor T cells to mature T cells, individual V, J and C gene segments are rearranged by somatic recombination to produce a functional TRA locus. Transcription and splicing produce a pre-mRNA and then mRNA for the complete VJC transcript sequence (not shown). In the modified 10x protocol, mRNA (including TRA mRNA) is converted to cDNA. TRA cDNA is then amplified using a nested PCR design. The forward primers from the 10x protocol were left unchanged (v2 protocol shown). In the first cycle, the forward primer (TRA Forward 1) primes off the Illumina read 1 (R1) sequencing adapter that is incorporated during generation of cDNA. In the second cycle, the identical forward primer (TRA Forward 2) again primes off the R1 sequence. The first reverse primer (TRA Reverse 1, Outer) primes off the constant (C) region gene segment. The second reverse primer (TRA Reverse 2, Inner) similarly primes off the C region at an inner, 5’ position relative to the outer primer. The α chain primer design was based off of a dog TCR α rearranged partial mRNA (GenBank: M97511.1) which was extended to include (from 3’ to 5’) the R1 adaptor, 10x cell barcode, UMI, TSO, V, J, and C gene segments. The constructed cDNA sequence was then used as input to primer3plus (4.0), with forward primers provided as described above, and a target region for reverse primer specified in the C region. The product of the first (outer) design was used as input for the second (inner) design.

Suppl Figure 2. Representative gel image of the final TCR amplification product

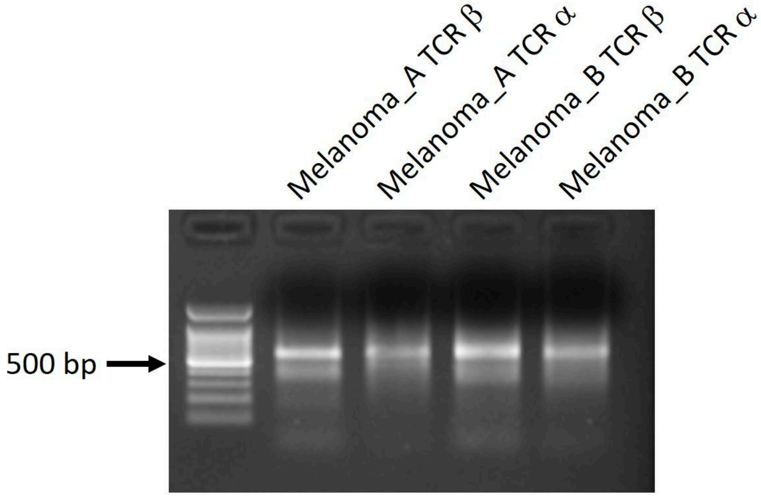

Gel electrophoresis result visualizing product of TCR-α (TRA) and TCR-β (TRB) nested PCR amplification for two representative samples (Melanoma_A_PBMC and Melanoma_B_PBMC) using custom primers (**Suppl Table 1**) and a modified Chromium 10x protocol (Methods). Gel bands of the expected size (~650bp) were observed for all reactions.

Suppl Figure 3. Representative analytic traces of the sequencing libraries prepared from TCR amplification products by fragment analyzer.

a) Melanoma_A_PBMC TCR α chain

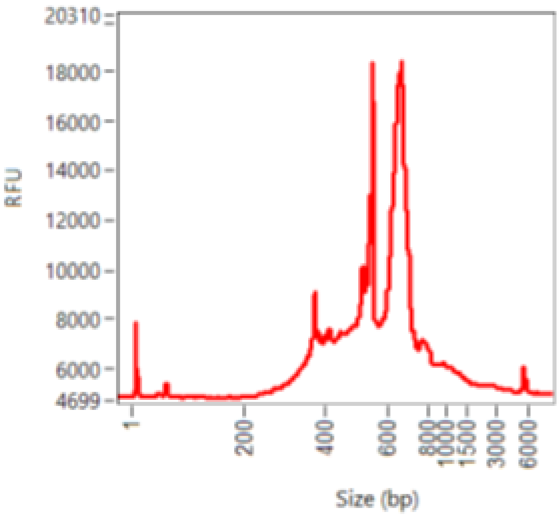

b) Melanoma_A_PBMC TCR β chain

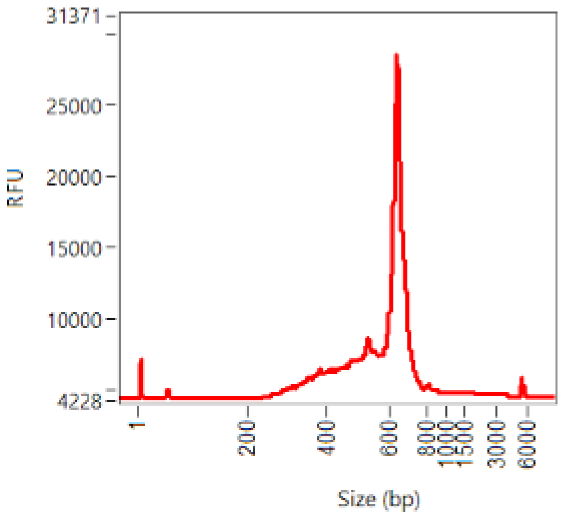

c) Melanoma_B_PBMC TCR α chain

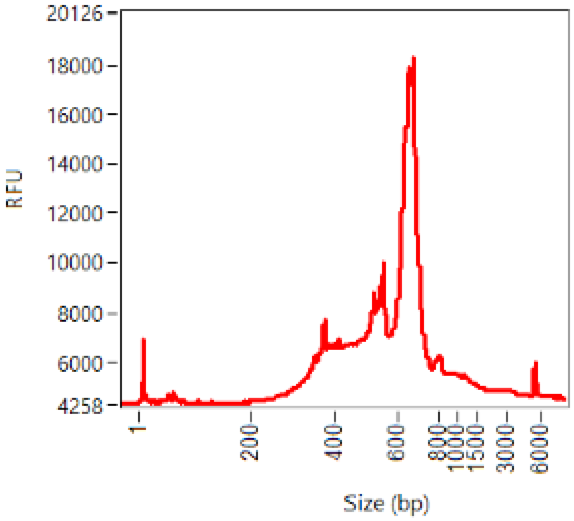

d) Melanoma_B_PBMC TCR β chain

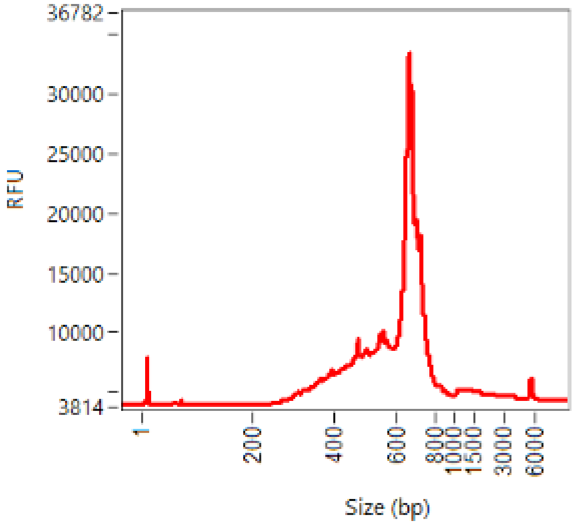

Sequencing libraries were constructed from the purified TCR amplification products and analyzed on the Agilent Fragment Analyzer using the NGS Fragment kit (DNF-473-500). The traces show representative scTCR libraries for (a) Melanoma_A_PBMC α chain, (b) Melanoma_A_PBMC β chain, (c) Melanoma_B_PBMC α chain, and (d) Melanoma_B_PBMC β chain

Suppl Figure 4. Representative analytic traces of the expression libraries by Fragment Analyzer.

a) Melanoma_A scRNA library, final product

b) Melanoma_B scRNA library, final product

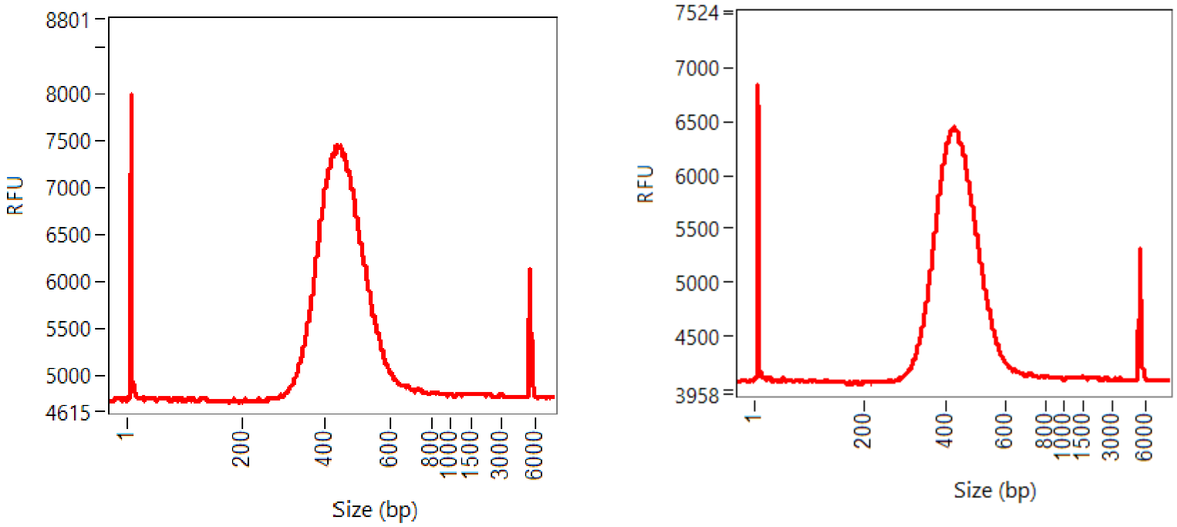

The final purified 5’ gene expression (scRNA) libraries were analyzed on an Agilent Fragment Analyzer using the NGS Fragment kit (DNF-473-500). The traces show representative gene expression libraries for (a) Melanoma_A_PBMC and (b) Melanoma_B_PBMC.

Suppl Figure 5. Aggregate VJ Gene Segment Usage for dog T-cell receptor α chain

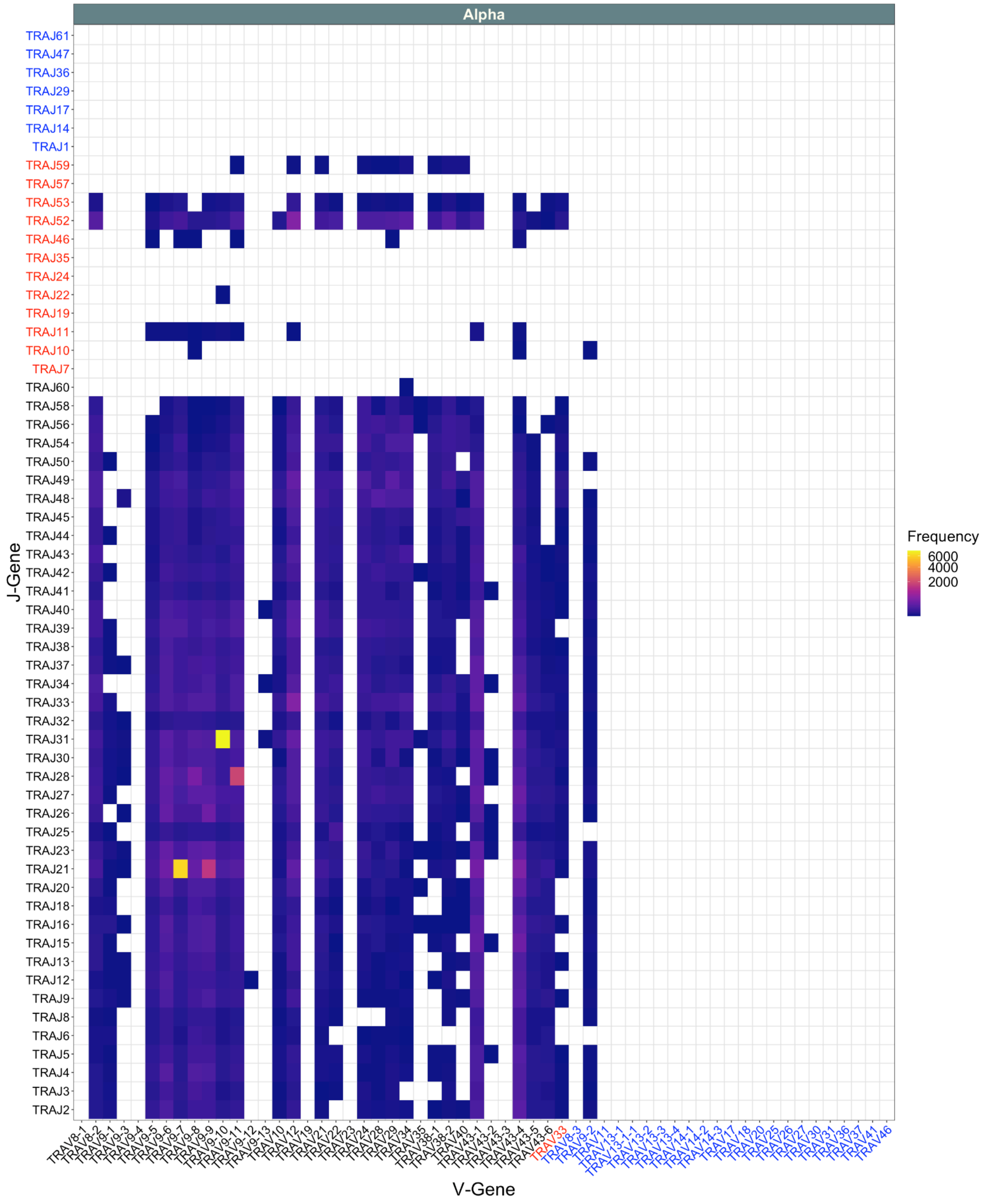

The VJ gene combinations identified in samples for all dogs (n = 16; normal LN, normal PBMC, melanoma PBMC, lymphoma LN) are plotted along with their observed cell barcode counts (Frequency) for the TRA chain. The corresponding VJ gene usage for the TRB chain is shown in **Figure 2**. Text in black indicates a functional annotation according to IMGT; blue indicates a pseudogene; red indicates a gene segment that has an ORF but also a defect in the splicing sites, recombination signals and/or regulatory elements or other features disqualifying a functional annotation.

Suppl Figure 6. Sequence analysis of TRAV9-2 pseudogene

a

```
IMGT000004|TRAV9-2*01|Canis        AATTGTACTTACTGAATGTCAGGGTATCCTGTCCTTTTCTGGTGTGT-CAGTATCTGGGG179
Normal_B_PBMC|clonotype3371        AATTGTACTTACTCAACGTCAGGGTATCCTGTCCTTTTCTGGTATGTCCAGTATCTGGGG180
Normal_A_PBMC|clonotype134         AATTGTACTTACTCAACGTCAGGGTATCCTGTCCTTTTCTGGTATGTCCAGTATCTGGGG180
Normal_C_LN|clonotype1431          AATTGTACTTACTCAACGTCAGGGTATCCTGTCCTTTTCTGGTATGTCCAGTATCTGGGG180
Melanoma_B_PBMC|clonotype956       AATTGTACTTACTCAACGTCAGGGTATCCTGTCCTTTTCTGGTATGTCCAGTATCTGGGG180
Normal_E_LN|clonotype1418          AATTGTACTTACTCAACGTCAGGGTATCCTGTCCTTTTCTGGTATGTCCAGTATCTGGGG180
Melanoma_A_PBMC|clonotype616       AATTGTACTTACTCAACGTCAGGGTATCCTGTCCTTTTCTGGTATGTCCAGTATCTGGGG180
PTCL_NOS_LN|clonotype332           AATTGTACTTACTCAACGTCAGGGTATCCTGTCCTTTTCTGGTATGTCCAGTATCTGGGG180
Normal_D_LN|clonotype164           AATTGTACTTACTCAACGTCAGGGTATCCTGTCCTTTTCTGGTATGTCCAGTATCTGGGG180
Melanoma_C_PBMC|clonotype322       AATTGTACTTACTCAACGTCAGGGTATCCTGTCCTTTTCTGGTATGTCCAGTATCTGGGG180
Melanoma_D_PBMC|clonotype403       AATTGTACTTACTCAACGTCAGGGTATCCTGTCCTTTTCTGGTATGTCCAGTATCTGGGG180
                                   ************* ** ************************** *** ************
```

b

```
>IMGT000004|TRAV9-2|Canis lupus familiaris_boxer|L-PART1+V-EXON
atg aag tgt tct cca ggg atc gtg att gtc cta ttc tta atg ctt gga caa acc cat gga
 M   K   C   S   P   G   I   V   I   V   L   F   L   M   L   G   Q   T   H   G  20
aac tca gtg aac cag act gaa ggc cag gtg acc gtc tca gaa gag gct tcc ttc aca atg
 N   S   V   N   Q   T   E   G   Q   V   T   V   S   E   E   A   S   F   T   M  40
aat tgt act tac tga atg tca ggg tat cct gtc ctt ttc tgg tgt gtc agt atc tgg gga
 N   C   T   Y   *   M   S   G   Y   P   V   L   F   W   C   V   S   I   W   G  60
atg gtc cac agc tcc tcc tga aag cat cag gag aca agg aga agg gaa gta aca aag ggt
 M   V   H   S   S   S   *   K   H   Q   E   T   R   R   R   E   V   T   K   G  80
ttg aag cca ctt tgg aca gtt cat cca aat cct tcc act tga aga aag gct cac tgc aag
 L   K   P   L   W   T   V   H   P   N   P   S   T   *   R   K   A   H   C   K  100
tgt cag act cag ctg tgt act act gtg tca tga gtg
 C   Q   T   Q   L   C   T   T   V   S   *   V                                 112
```

c

```
>Normal_A_PBMC|clonotype134
atg aag tgt tct cca ggg atc gtg att gtc cta ttc tta atg ctt gga caa acc cat gga
 M   K   C   S   P   G   I   V   I   V   L   F   L   M   L   G   Q   T   H   G  20
aac tca gtg aac cag act gaa ggc cag gtg acc ctc tca gaa gag gct tcc ttg act atg
 N   S   V   N   Q   T   E   G   Q   V   T   L   S   E   E   A   S   L   T   M  40
aat tgt act tac tca acg tca ggg tat cct gtc ctt ttc tgg tat gtc cag tat ctg ggg
 N   C   T   Y   S   T   S   G   Y   P   V   L   F   W   Y   V   Q   Y   L   G  60
aat ggt cca cag ctc ctc ctg aaa gca tca gga gac aag gag aag gga agt aac aaa ggg
 N   G   P   Q   L   L   L   K   A   S   G   D   K   E   K   G   S   N   K   G  80
ttt gaa gcc act ttg gac agc tca tcc aaa tcc ttc cac ttg aag aaa ggc tca ctg cac
 F   E   A   T   L   D   S   S   S   K   S   F   H   L   K   K   G   S   L   H  100
gtg tca gac tca gct gtg tac tac tgt gtc atg
 V   S   D   S   A   V   Y   Y   C   V   M                                     111
```

(a) A Clustal Omega multisequence alignment shows 10 randomly selected clonotypes from 10 different dogs compared to the reference sequence for amino acid positions 41 to 60 of the TRAV9-2 gene segment. A stop codon (TGA) and single base frameshift deletion, as annotated in IMGT, and unique to the reference sequence, are highlighted in red. Four consistent nucleotide differences (3 SNVs and 1 insertion) between clonotypes and reference are indicated along the bottom. (b) The reference TRAV9-2 coding sequence is shown with a stop codon (TGA) at amino acid position 45 and additional stop codons resulting from frameshift deletion are highlighted in red. (c) A representative TRAV9-2 clonotype coding sequence is shown with the alterations that correct nonsense and frameshift mutations observed in the reference.

Suppl Figure 7. Sequence analysis of TRBV19 pseudogene

a

Normal_E_PBMC clonotype73

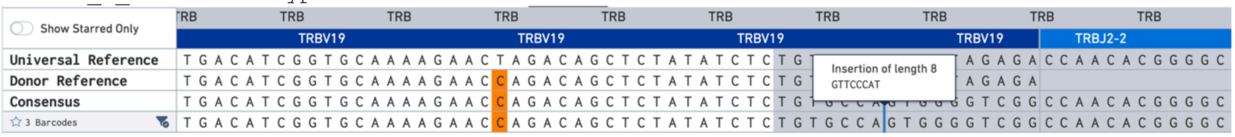

b

```
>IMGT000005|TRBV19|Canis lupus familiaris_boxer|P|L-PART1+V-EXON
atg ggt aac cag gtg atc tgc tgt gtg gcc ctt tgt ctc ctc gga gca gga aca gca agt
 M   G   N   Q   V   I   C   C   V   A   L   C   L   L   G   A   G   T   A   S  20
ggt gga atc act cag acc ccc aaa tat ttg ttc aga gag gaa gga cga ggt gtg act ctg
 G   G   I   T   Q   T   P   K   Y   L   F   R   E   E   G   R   G   V   T   L  40
gaa tgt gaa cag gat ttt aat cat gac tct atg tac tgg tac cga caa gac cca ggg caa
 E   C   E   Q   D   F   N   H   D   S   M   Y   W   Y   R   Q   D   P   G   Q  60
ggg ctg aga ctg atc tac tac tcg ctg gta gaa aat gat gct cag aaa gga gac ata cct
 G   L   R   L   I   Y   Y   S   L   V   E   N   D   A   Q   K   G   D   I   P  80
gaa ggc tac agt gcc tct cgg atg aag aag gca ttc ttc tct ctc acc atg aca tcg gtg
 E   G   Y   S   A   S   R   M   K   K   A   F   F   S   L   T   M   T   S   V  100
caa aag aac tag aca gct cta tat ctc tgt gcc agt ggt aga
 Q   K   N   *   T   A   L   Y   L   C   A   S   G   R                          114
```

c

```
>Normal_E_PBMC|clonotype73
atg ggt aac cag gtg atc tgc tgt gcg gcc ctt tgt ctc ctc gga gca gga aca gca agt
 M   G   N   Q   V   I   C   C   A   A   L   C   L   L   G   A   G   T   A   S  20
ggt gga atc act cag acc ccc aaa tat ttg ttc aga gag aaa gga cga ggc gtg act ctg
 G   G   I   T   Q   T   P   K   Y   L   F   R   E   K   G   R   G   V   T   L  40
gaa tgt gaa cag gat ttt aat cat gac tct atg tac tgg tac cga caa gac cca ggg caa
 E   C   E   Q   D   F   N   H   D   S   M   Y   W   Y   R   Q   D   P   G   Q  60
ggg ctg aga ctg atc tac tac tcg ctg gta gaa aat gat gct cag aaa gga gac ata cct
 G   L   R   L   I   Y   Y   S   L   V   E   N   D   A   Q   K   G   D   I   P  80
gaa ggc tac agt gcc tct cgg atg aag aag gca ttc ttc tct ctc acc atg aca tcg gtg
 E   G   Y   S   A   S   R   M   K   K   A   F   F   S   L   T   M   T   S   V  100
caa aag aac cag aca gct cta tat ctc tgt gcc agt tcc cat gtg ggg tcg
 Q   K   N   Q   T   A   L   Y   L   C   A   S   S   H   V   G   S              117
```

(a) A portion of a representative clonotype sequence is shown for Normal_E_PBMC. A single base substitution (T->C) is highlighted in orange and shown to be present in the germline (Donor Reference) of this dog. Note, an insertion (GTTCCCAT), likely related to end-joining, is also shown with a blue bar. (b) The TRBV19 reference coding sequence is shown with stop codon (TAG) at amino acid position 104, highlighted in red. (c) A representative TRBV19 clonotype coding sequence is shown with the T->C alteration that corrects the nonsense mutation observed in the reference to a (CAG) Q amino acid.

Suppl Figure 8. Sequence analysis of TRBJ1-3 pseudogene.

a

Tzone_LSA_LN clonotype1

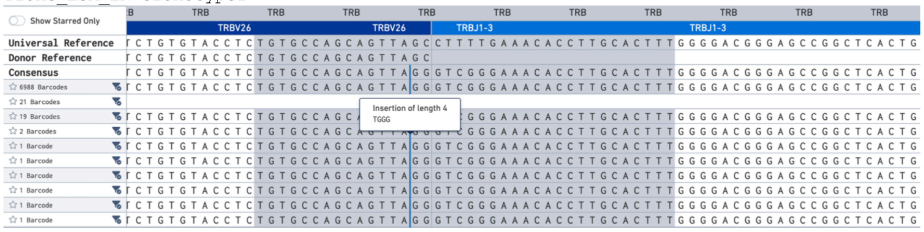

b

```
>IMGT000005|TRBV26/TRBJ1-3|Canis lupus familiaris_boxer|L-PART1+V-EXON+J-REGION
atg agc aac agg ttg ctc tgc tgt gtt gtc att tgt ctt gtc aaa gta ggt ctc aag gat
 M   S   N   R   L   L   C   C   V   V   I   C   L   V   K   V   G   L   K   D  20
gct ctg gtc aat cag ttc cca aga cat agg atc ttg ggg aca gga aag aaa tta acc cta
 A   L   V   N   Q   F   P   R   H   R   I   L   G   T   G   K   K   L   T   L  40
cag tgt ttg cag gat atg aat cat gtt tca atg ttc tgg tat cgc caa gac cca gga ttt
 Q   C   L   Q   D   M   N   H   V   S   M   F   W   Y   R   Q   D   P   G   F  60
ggg cta cag ctg atc tac tac tca act ggt act gac aac ttt gaa aaa gga gat gcc cct
 G   L   Q   L   I   Y   Y   S   T   G   T   D   N   F   E   K   G   D   A   P  80
gag ggg tat gat gtc tct cga aat gag ctg aaa tct ttt ccc ctg acc ctg gtc tct gcc
 E   G   Y   D   V   S   R   N   E   L   K   S   F   P   L   T   L   V   S   A  100
agc acc aac cag aca tct gtg tac ctc tgt gcc agc agt tag cct ttt gaa aca cct tgc
 S   T   N   Q   T   S   V   Y   L   C   A   S   S   *   P   F   E   T   P   C  120
act ttg ggg acg gga gcc ggc tca ctg ttg tag
 T   L   G   T   G   A   G   S   L   L   *                                      131
```

c

```
> Tzone_LSA_LN|clonotype1
atg agc aac agg ttg ctc tgc tgt gtt gtc att tgt ctt gtc aaa gta ggt ctc aag gat
 M   S   N   R   L   L   C   C   V   V   I   C   L   V   K   V   G   L   K   D  20
gct ctg gtc aat cag ttc cca aga cat agg atc ttg ggg aca gga aag aaa tta acc cta
 A   L   V   N   Q   F   P   R   H   R   I   L   G   T   G   K   K   L   T   L  40
cag tgt ttg cag gat atg aat cat gtt tca atg ttc tgg tat cgc caa gac cca gga ttt
 Q   C   L   Q   D   M   N   H   V   S   M   F   W   Y   R   Q   D   P   G   F  60
ggg cta cag ctg atc tac tac tca act ggt act gac aac ttt gaa aaa gga gat gcc cct
 G   L   Q   L   I   Y   Y   S   T   G   T   D   N   F   E   K   G   D   A   P  80
gag ggg tat gat gtc tct cga aat gag ctg aaa tct ttt ccc ctg acc ctg gtc tct gcc
 E   G   Y   D   V   S   R   N   E   L   K   S   F   P   L   T   L   V   S   A  100
agc acc aac cag aca tct gtg tac ctc tgt gcc agc agt tat ggg ggg tcg gga aac acc
 S   T   N   Q   T   S   V   Y   L   C   A   S   S   Y   G   G   S   G   N   T  120
ttg cac ttt ggg gac ggg agc cgg ctc act gtt gta
 L   H   F   G   D   G   S   R   L   T   V   V                                  132
```

d

```
IMGT000005|TRBJ1-3*01|Canis      ---------CTTTTGAAACACCTTGCACTTTGGGGACGGGAGCCGGCTCACTGTTGTAG   50
Melanoma_B_PBMC|clonotype65      ---------GACGGGCGGTGGCTTGCACTTTGGGGACGGGAGCCGGCTCACTGTTGTA-   49
Normal_D_PBMC|clonotype13        CGGACTACAGGTAACTAACACCTTGCACTTTGGGGACGGGAGCCGGCTCACTGTTGTA-   58
Normal_E_PBMC|clonotype6         -----ATAGTGGGCTTGACACCTTGCACTTTGGGGACGGGAGCCGGCTCACTGTTGTA-   53
Normal_B_LN|clonotype4           ------------TATGGACACCTTGCACTTTGGGGACGGGAGCCGGCTCACTGTTGTA-   46
Normal_C_PBMC|clonotype36        ---------GGGTAGCAACACCTTGCACTTTGGGGACGGGAGCCGGCTCACTGTTGTA-   49
PTCL_NOS_LN|clonotype38          ---------ACACGAGAACACCTTGCACTTTGGGGACGGGAGCCGGCTCACTGTTGTA-   49
Normal_A_LN|clonotype16          ---------GGACGTAGAAACCTTGCACTTTGGGGACGGGAGCCGGCTCACTGTTGTA-   49
Melanoma_D_PBMC|clonotype3       ---------ATTAGGAAACACCTTGCACTTTGGGGACGGGAGCCGGCTCACTGTTGTA-   49
Tzone_LSA_LN|clonotype1          ---------GTCGGGAAACACCTTGCACTTTGGGGACGGGAGCCGGCTCACTGTTGTAG   50
Melanoma_C_PBMC|clonotype18      ACACTCAGGGGGGGAAGACACCTTGCACTTTGGGGACGGGAGCCGGCTCACTGTTGTA-   58
Melanoma_A_PBMC|clonotype23      -----------GGGAAAGCACCTTGCACTTTGGGGACGGGAGCCGGCTCACTGTTGTA-   47
                                                      *************************************
```

(a) A portion of the dominant clonotype sequence is shown for TZone_LSA_LN revealing a 4bp insertion and 5 other single base pair changes closely flanking the VJ joining boundary. (b) The TRBV26/TRBJ1-3 reference coding sequence is shown. Without modifications these reference sequences include a stop (TAG) at the end of the V segment (first red highlight) and the entire J segment (blue bases) is out of frame. A stop early in the TRBJ1-3 pseudogene is avoided with this frame (second red highlight) but another stop is introduced at the end of the J gene (third red highlight). (c) The dominant TRBV26/TRBJ1-3 clonotype coding sequence from TZone_LSA_LN is shown with no stop codons and the J-gene portion is now in the correct frame. (d) A Clustal Omega multisequence alignment shows the dominant TZone_LSA_LN clonotype along with 10 randomly selected clonotypes from 10 different dogs compared to the reference sequence for the first ~50 bp of the TRBJ1-3 gene segment. In all cases, unique sequence changes introduced during VJ joining eliminate the stop codon from the beginning of TRBJ1-3 while preserving the correct frame.

Suppl Figure 9. Median TRA/TRB VJ gene segment usage

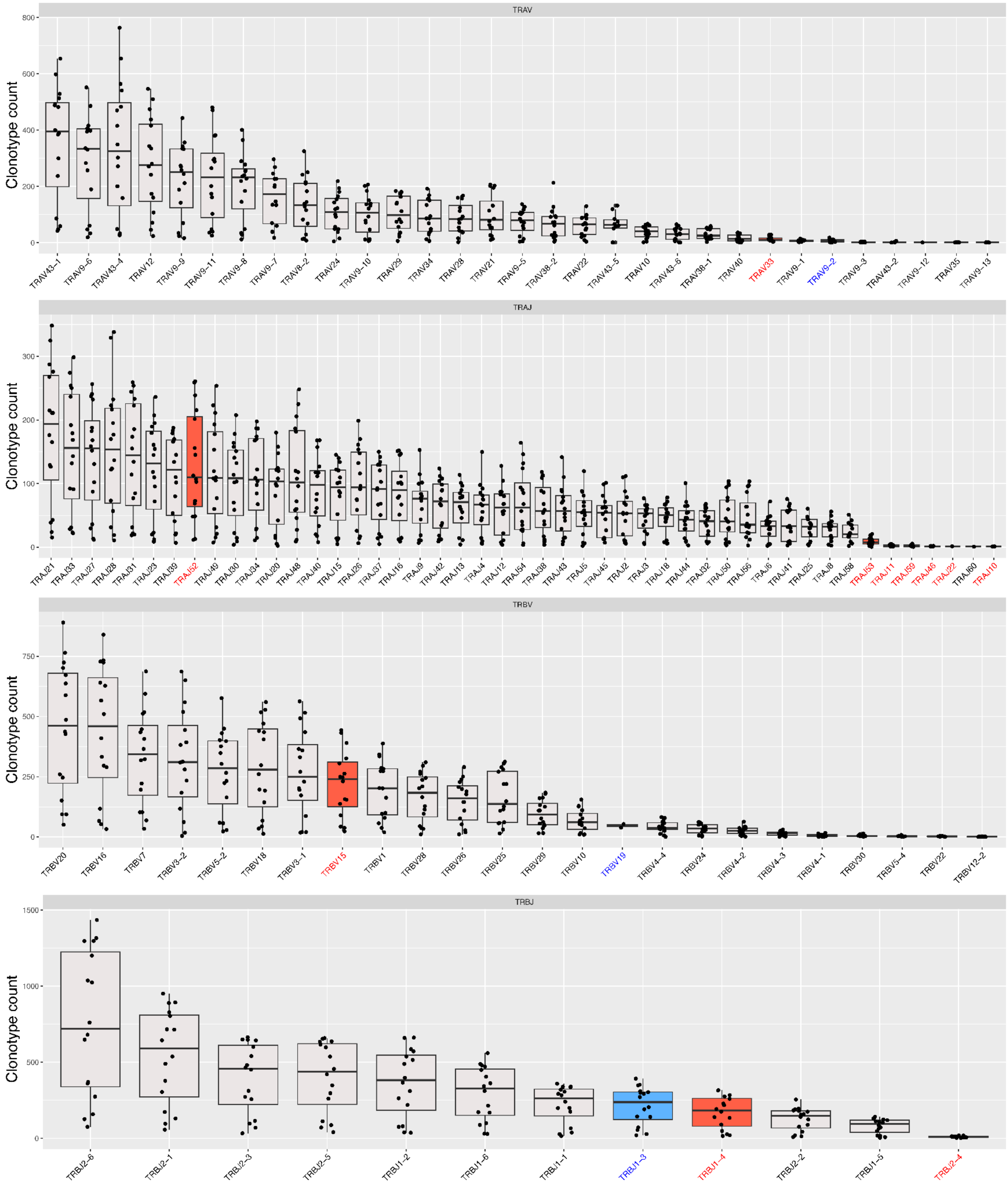

Box and whisker plots showing the median number of unique clonotypes making use of each TRAV, TRAJ, TRBV, and TRBJ gene segment for all 16 samples are shown. Gene segment boxes and labels are colored according to their IMGT annotation (functional, gray; non-functional ORF, red; pseudogene, blue). Note, only gene segments observed in at least one clonotype in at least one cell are plotted (31/58 TRAV, 47/59 TRAJ, 24/36 TRBV, and 12/12 TRBJ total gene segments). The box plot shows median (center line), interquartile range (IQR; box limits), largest value at most 1.5 times IQR (upper whisker), smallest value at most 1.5 times IQR (lower whisker) and outliers (points beyond whiskers).

Suppl Figure 10. Length distributions of dog TCR alpha chain and beta chain

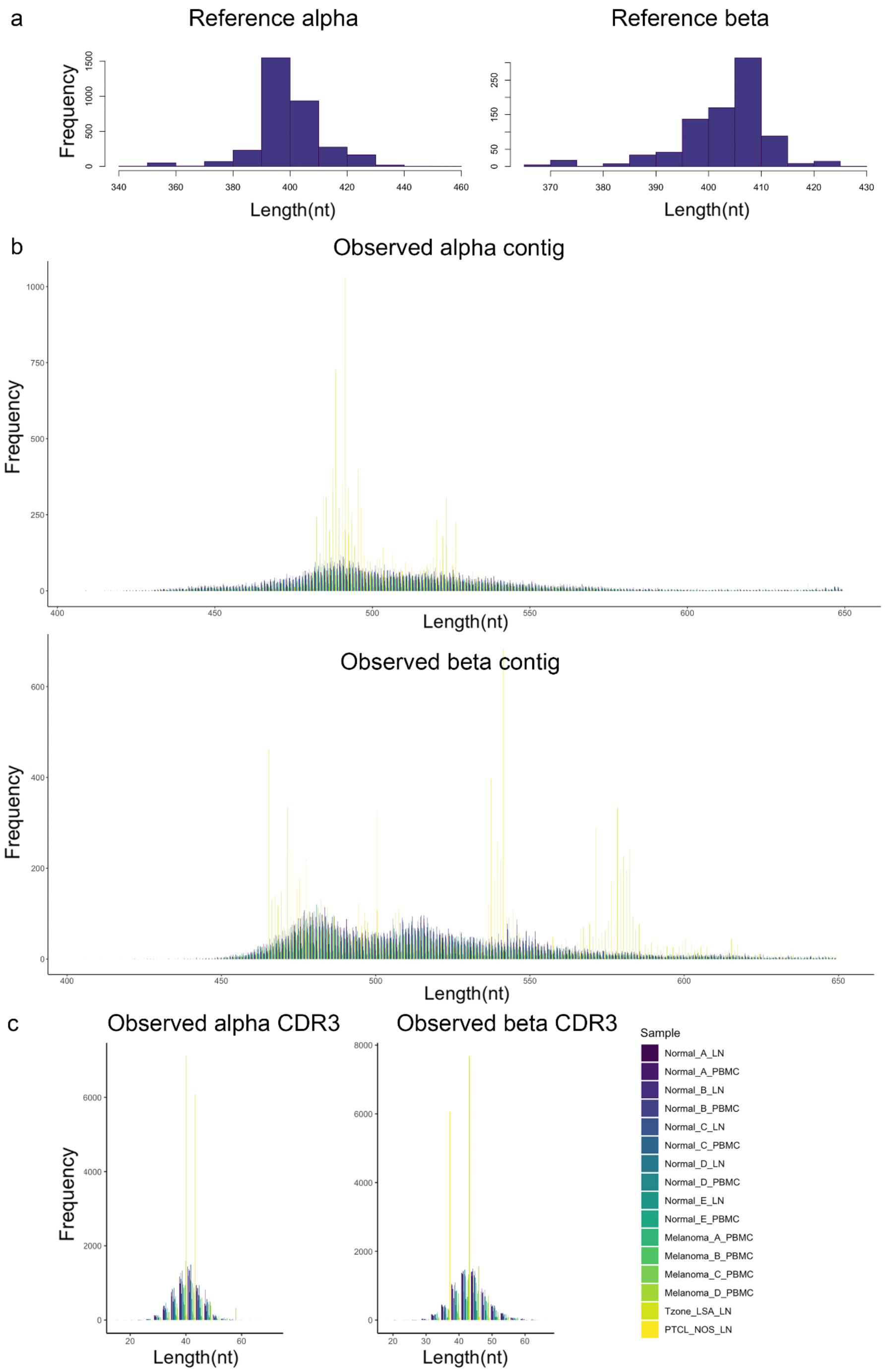

(a) Distributions of reference lengths for TCR alpha and beta chains. The length of each gene (TRA-V/J, TRB-V/D/J) was inferred from IMGT reference fasta sequences. The reference length for TCR chain was calculated as the sum of TRAV and TRAJ gene segment lengths for alpha chain, and the sum of TRBV, TRBD, and TRBJ gene segment lengths for beta chain. (b) Distribution of observed contig lengths for TCR alpha and beta chains. (c) Distribution of observed CDR3 lengths for TCR alpha and beta chains. Both observed contig and CDR3 lengths were retrieved from cellranger vdj results. All lengths are reported in nucleotides (nt).

Suppl Figure 11. Single cell clonotype distribution for TRA and TRB chains for all samples after downsampling

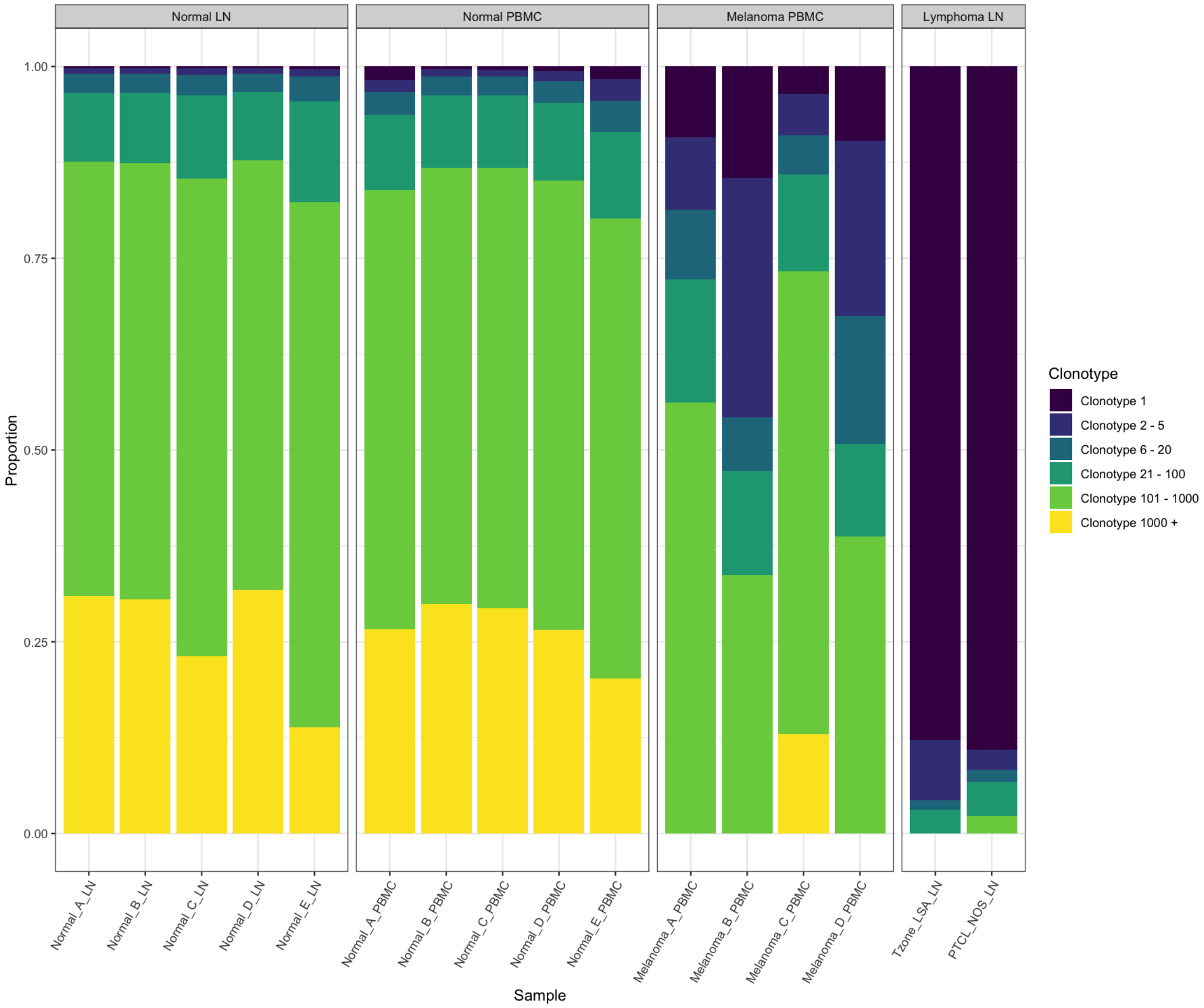

The proportion of total barcodes, for all clonotypes, is shown for the lymph node aspirates (Normal_LN) and PBMCs (Normal_PBMC) from five healthy dogs, PBMCs from four dogs with melanoma (Melanoma_PBMC) and lymph node aspirates from two dogs with T cell lymphoma (Lymphoma_LN). Proportion was estimated by downsampling the number of cells to the smallest number of cells with a detected clonotype (Melanoma_A_PBMC, n=1850), calculating the fraction of cells in each bin (Clonotype 1, Clonotype 2 - 5, etc where the clonotypes are sorted in descending order of cell counts), repeating 100 times, and calculating the average. The healthy normal samples are characterized by highly diverse clonotypes, with even the most frequent clonotype observed in only a very small proportion of cells. The melanoma PBMC cases are characterized by a small number of dominant clonotypes with higher frequency. The T cell lymphoma cases are characterized by one dominant clonotype in each case.

Suppl Figure 12. Single cell clonotype distribution for TRA and TRB chains for all samples for CD4+ and CD8+ T cell types

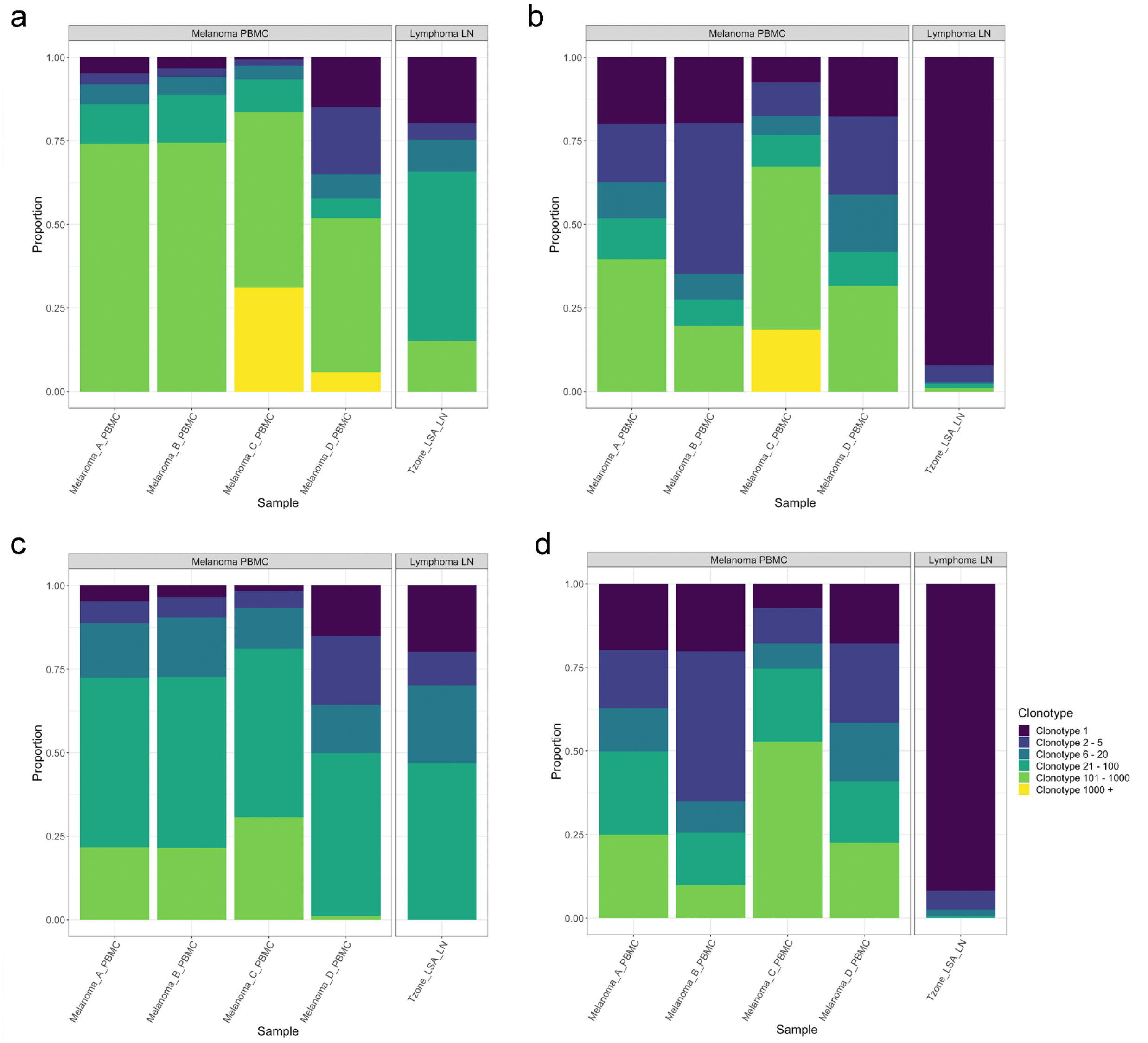

The proportion of total barcodes, for all clonotypes, is shown for the PBMCs from four dogs with melanoma and lymph node aspirate from one dog with T cell lymphoma for CD4+ cells (panels a, c) and CD8+ cells (panels b, d) according to singleR cell typing. In panel a and b, the proportion was estimated by calculating the fraction of cells in each bin (Clonotype 1, Clonotype 2 - 5, etc where the clonotypes are sorted in descending order of cell counts). In panel c and d, the proportion was estimated by downsampling the number of cells to the smallest number of cells with a detected clonotype (Tzone_LSA_LN, n=158 for CD4+ and Melanoma_A_PBMC, n=773 for CD8+), calculating the fraction of cells in each bin, repeating 100 times, and calculating the average. For the CD8+ subset of cells (panels b and d), the T cell lymphoma (Tzone_LSA_LN) is characterized by one dominant clonotype. The melanoma PBMC cases are characterized by a small number of dominant clonotypes with higher frequency. This is similar to the pattern observed for all T cells (**Figure 4**). In contrast, for the CD4+ subset of cells (panels a and c) a greater diversity of clonotypes was observed for the T cell lymphoma sample and three of four melanoma samples. Melanoma_D_PBMC had similar patterns of CD4 and CD8 expansion.

Suppl Figure 13. Number and proportion of cells corresponding to the dominant clonotype for each cell type.

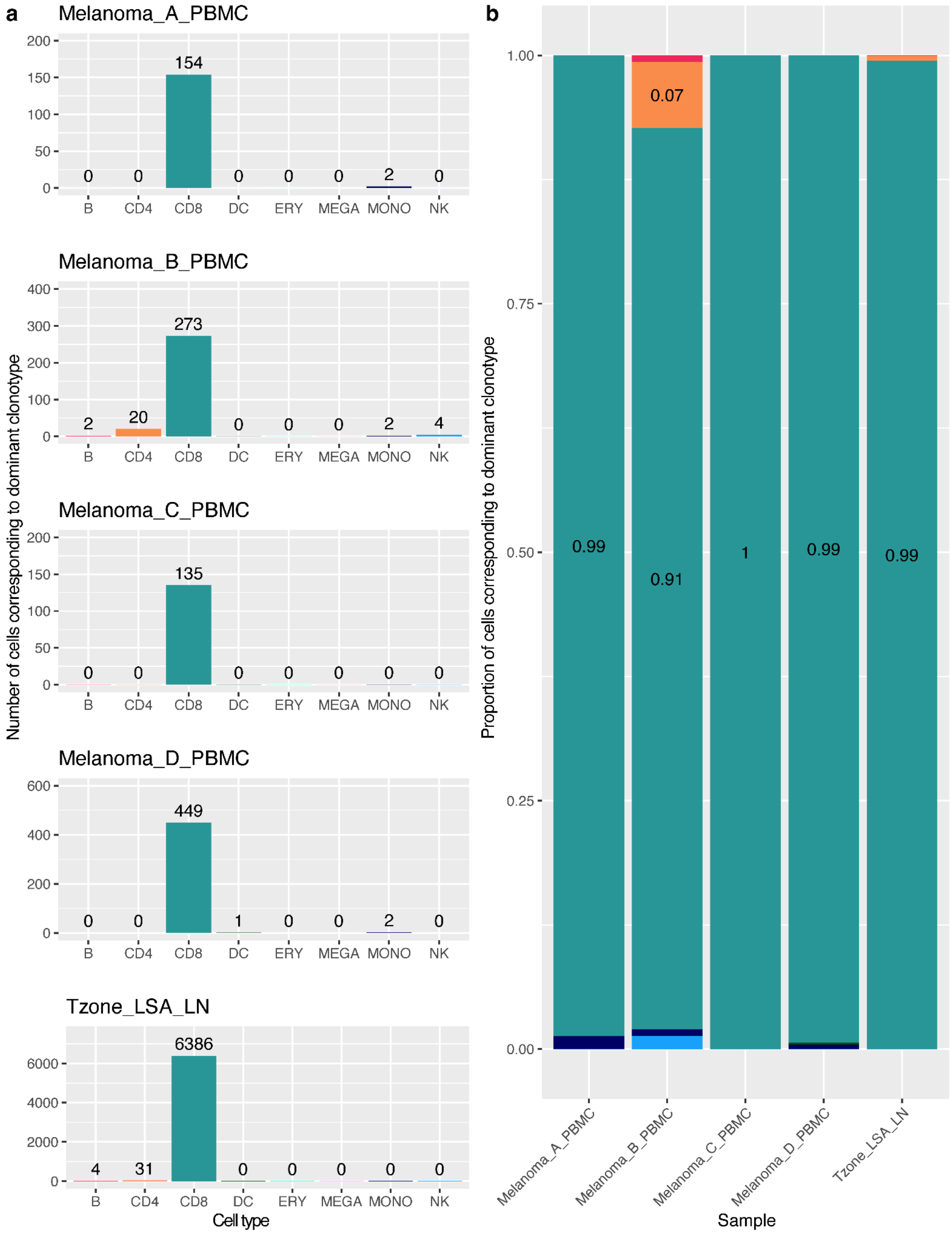

Plots show cell type assignment for cells corresponding to dominant clonotypes, in number (a) and proportion (b), for Melanoma_A_PBMC, Melanoma_B_PBMC, Meloma_C_PBMC, Melanoma_D_PBMC, and Tzone_LSA_LN. Dominant clonotype here is defined as the single most frequent clonotype in each sample. Cell types summarized include: B cell (B), CD4+ T cell (CD4), CD8+ T cell (CD8), Dendritic Cell (DC), erythrocyte (ERY), megakaryocyte (MEGA), monocyte (MONO), and Natural Killer cell (NK). Cells corresponding to dominant clonotypes in the scTCRseq experiment were almost exclusively assigned as T cells in scRNAseq experiment, but occasionally assigned as other cell types, potentially due to sample impurity (doublets) or uncertainty/errors in the cell assignment algorithm. Cells corresponding to dominant clonotypes were more often CD8+ than CD4+ T cells.

Suppl Figure 14. Expression of T cell activation and exhaustion markers in expanded clonotypes versus non-expanded clonotypes for Melanoma_A_PBMC, Melanoma_C_PBMC, Melanoma_D_PBMC, and Tzone_LSA_LN.

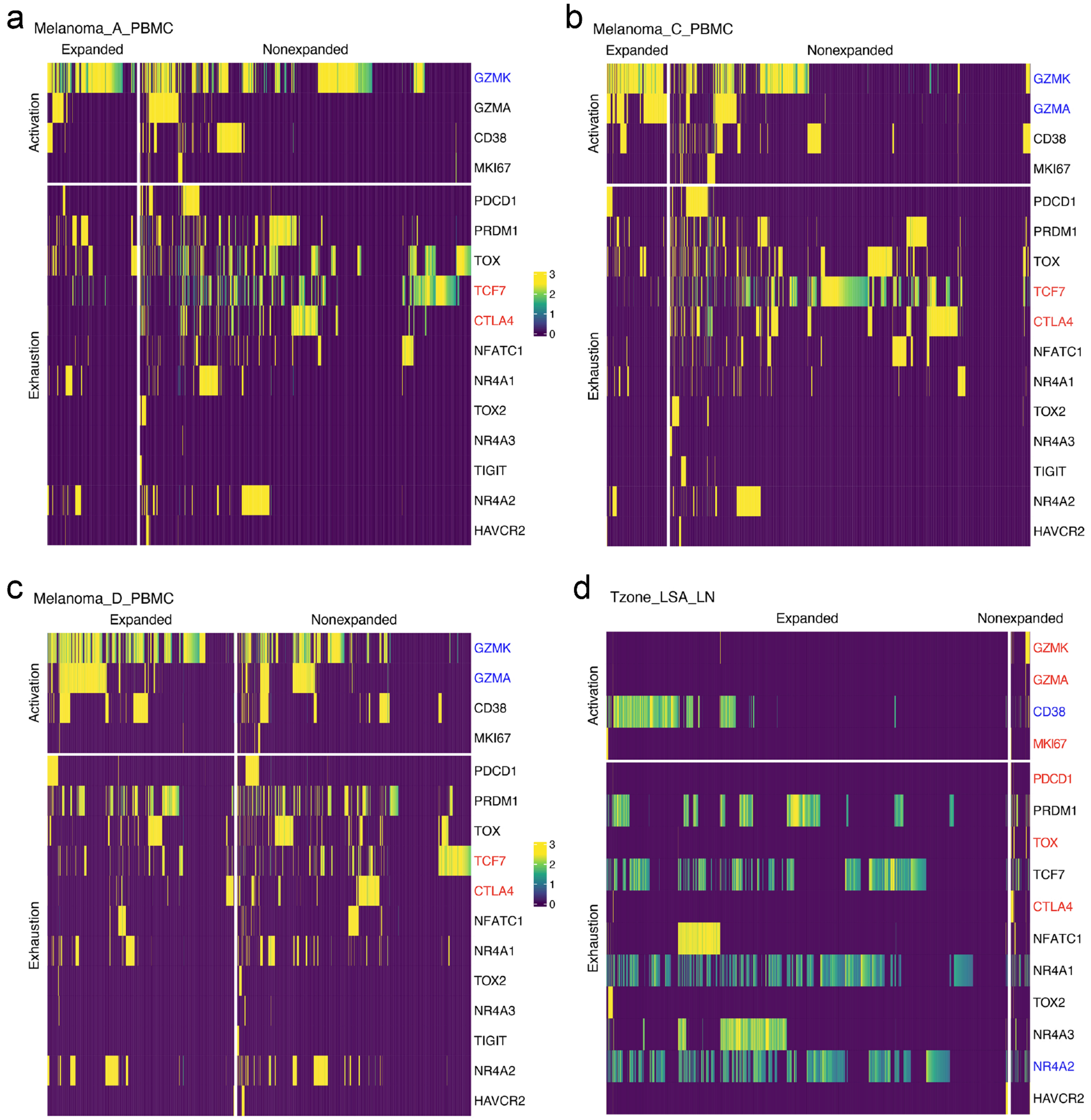

Each panel is a heatmap of single cell expression values ($\log_e(x + 1)$ normalized and scaled for all cells in the sample) for expanded CD8+ T cells vs non-expanded CD8+ T cells for known markers of T cell activation and exhaustion for (a) Melanoma_A_PBMC, (b) Melanoma_C_PBMC, (c) Melanoma_D_PBMC, and (d) Tzone_LSA_LN. Expanded cells are those with a clonotype frequency greater than 1%. Marker genes are colored blue if their expression was significantly increased or red if significantly decreased in expanded vs non-expanded (adjusted p-value < 0.05) (**Suppl Data 8**).

Suppl Figure 15. Expression of CD4, CD8, markers of effector memory T-cells, and markers of naive T-cells in scTCR dataset.

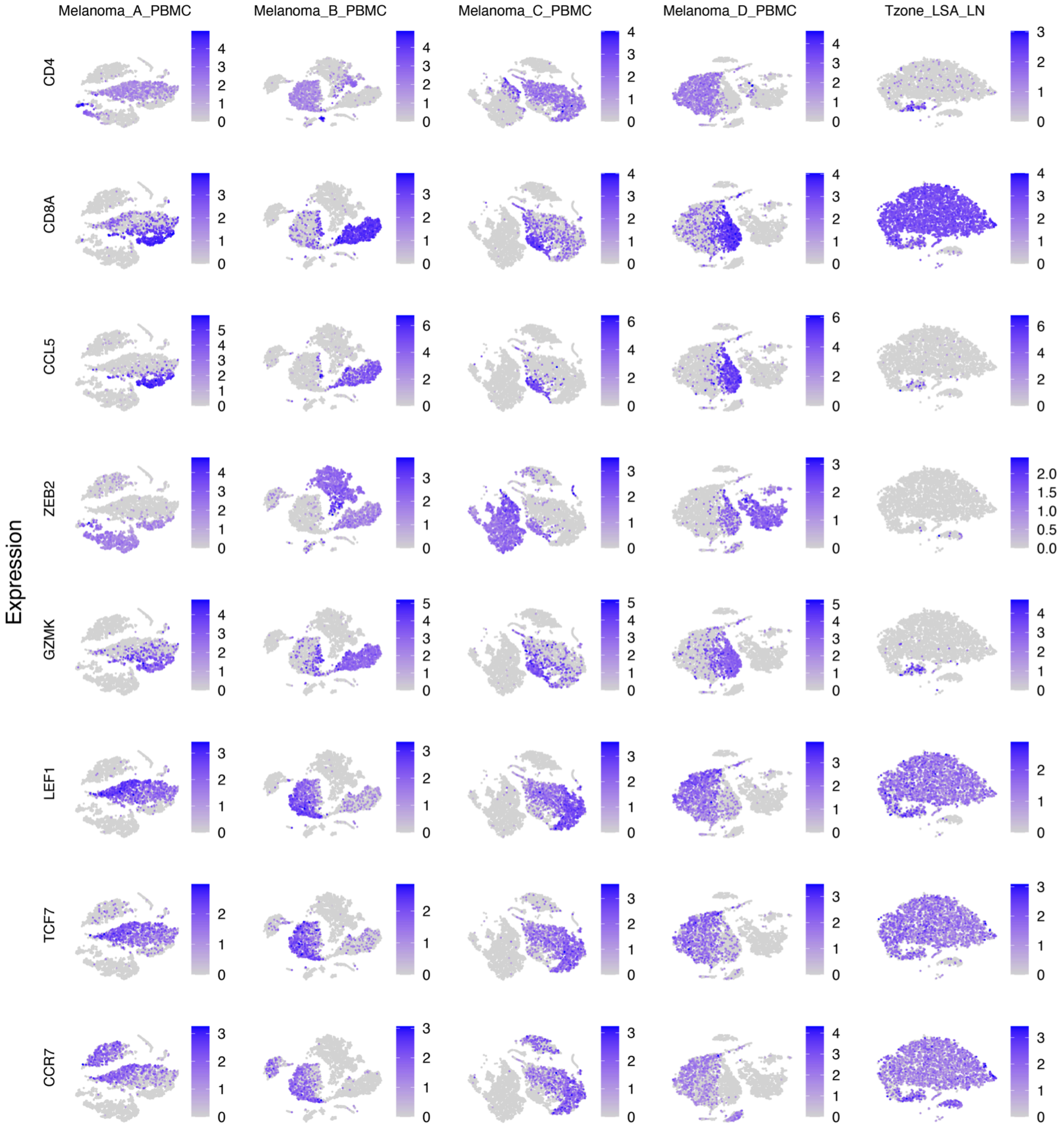

Expression of CD4, CD8, markers of effector memory T-cells (CCL5, ZEB2, GZMK) and markers of naive T-cells (LEF1, TCF7, CCR7) were projected onto t-SNE maps (**See Figure 6 for details**) for scTCR samples of dogs with melanoma or T zone lymphoma. Overall, for melanoma samples, the expanded CD8+ population (defined by dominant cluster in Figure 7D) mainly expressed effector memory markers, whereas the non-expanded, predominantly CD4+ population mainly expressed naive markers. For the T zone lymphoma sample, the expanded CD8+ population (presumed malignant clone) predominantly expressed naive markers. The small non-expanded, CD4+ cluster mainly expressed naive markers but also some effector memory.

Suppl Figure 16. Expression of CD4, CD8, markers of effector memory T-cells, and markers of naive T-cells in a subset of CD8+ T-cells that co-clusters with CD4+ T-cells

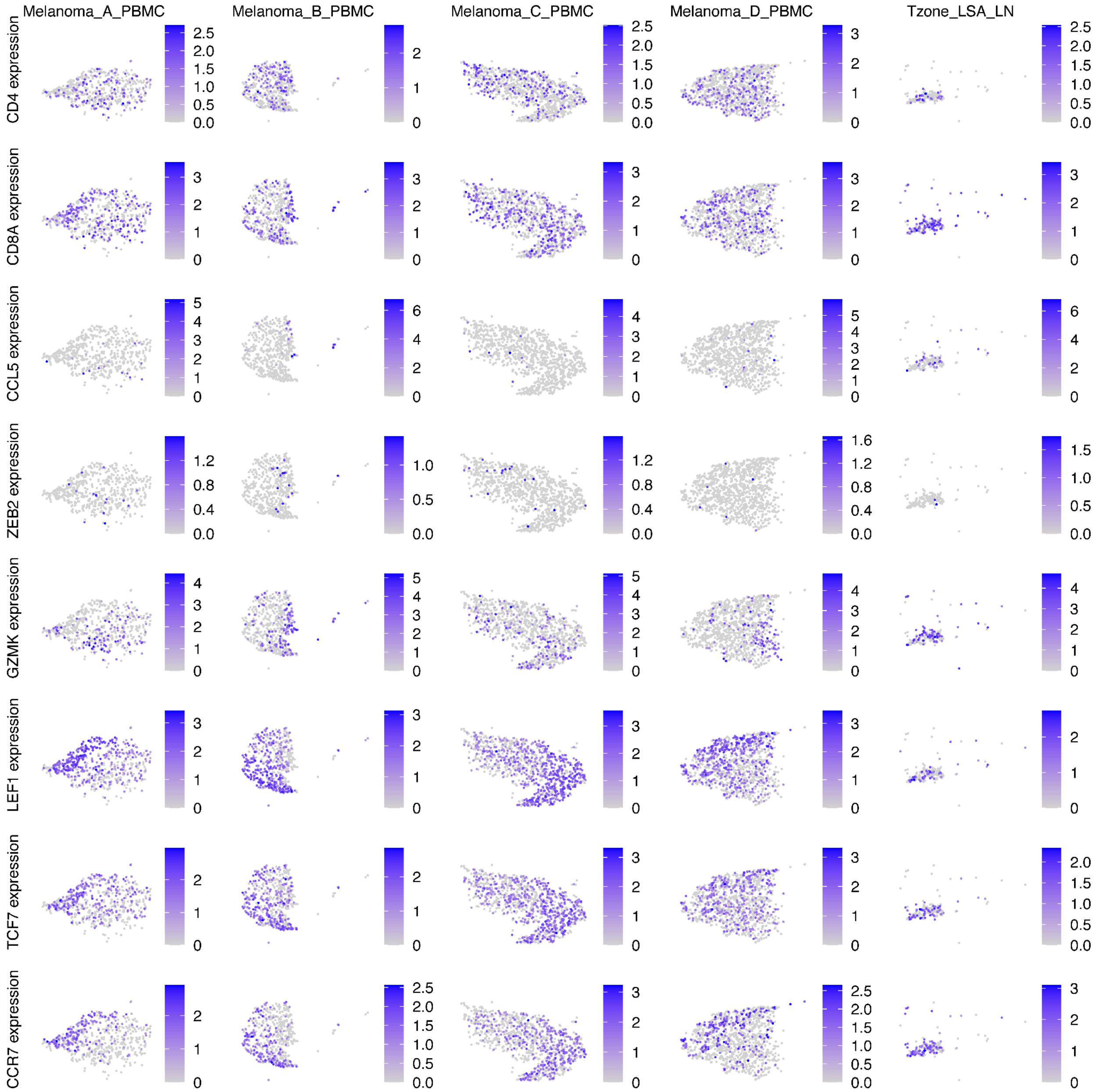

Expression of CD4, CD8, markers of effector memory T-cells (CCL5, ZEB2, GZMK) and markers of naive T-cells (LEF1, TCF7, CCR7) for a subset of CD8+ T-cells that co-cluster with CD4+ T-cells (defined by singleR cell typing) were projected onto t-SNE maps (See **Figure 6** for details) for scTCR samples of dogs with melanoma or T zone lymphoma. As expected, a subset of cells classified as CD8+ by singleR did not display CD8 expression. A portion of this CD8+ subset was also CD4+. The subset of CD8+ T-cells co-clustering with the CD4+ population primarily expressed naive markers and not effector memory markers.

Supplementary References

1. Rütgen, B. C. *et al.* Composition of lymphocyte subpopulations in normal canine lymph nodes. *Vet. Clin. Pathol.* **44**, 58–69 (2015).
2. Rissetto, K. C. *et al.* Cloning and expression of canine CD25 for validation of an anti-human CD25 antibody to compare T regulatory lymphocytes in healthy dogs and dogs with osteosarcoma. *Vet. Immunol. Immunopathol.* **135**, 137–145 (2010).
3. Pinheiro, D. *et al.* Phenotypic and functional characterization of a CD4(+) CD25(high) FOXP3(high) regulatory T-cell population in the dog. *Immunology* **132**, 111–122 (2011).
4. Sparger, E. E. *et al.* T Cell Immune Profiles of Blood and Tumor in Dogs Diagnosed With Malignant Melanoma. *Front Vet Sci* **8**, 772932 (2021).
5. Byrne, K. M., Kim, H. W., Chew, B. P., Reinhart, G. A. & Hayek, M. G. A standardized gating technique for the generation of flow cytometry data for normal canine and normal feline blood lymphocytes. *Vet. Immunol. Immunopathol.* **73**, 167–182 (2000).
