# Supplementary material for: Single-cell T-cell receptor repertoire profiling in dogs
Source: Commun Biol. 2024 Apr 22;7:484. doi: 10.1038/s42003-024-06174-w (PMC11035579; doi:10.1038/s42003-024-06174-w)
Supplement: Supplementary file 3 — Description of Additional Supplementary Files [file 42003_2024_6174_MOESM3_ESM.pdf]

Description of Additional Supplementary Files

File name: Supplementary Data 1
Description: Single cell V(D)J sequencing (scTCR) raw metrics (N=16 samples).

File name: Supplementary Data 2
Description: Single cell gene expression sequencing (scRNA) raw metrics (N=5 samples).

File name: Supplementary Data 3
Description: TCR clonotypes observed across all dogs.

File name: Supplementary Data 4
Description: Alternate consensus reference sequence for TRAV9-2.

File name: Supplementary Data 5
Description: Canine TRA/TRB VJ gene segment functional annotations from IMGT.

File name: Supplementary Data 6
Description: Alternate V gene alleles observed.

File name: Supplementary Data 7
Description: Alternate donor (germline) TRAV and TRBV sequences inferred by cell ranger.

File name: Supplementary Data 8
Description: Differential expression analysis of T cell activation and exhaustion markers in CD8+ T cells of expanded clonotypes versus non-expanded clonotypes.

File name: Supplementary Data 9
Description: VDJ reference sequences for use with cell ranger.

File name: Supplementary Data 10
Description: T cell phenotype markers investigated in human and dog.
