# Supplementary material for: Single-cell T-cell receptor repertoire profiling in dogs
Source: Commun Biol. 2024 Apr 22;7:484. doi: 10.1038/s42003-024-06174-w (PMC11035579; doi:10.1038/s42003-024-06174-w)
Supplement: Supplementary file 14 — Reporting Summary [file 42003_2024_6174_MOESM14_ESM.pdf]

nature portfolio

Corresponding author(s): Jeffrey Bryan, Obi Griffith

Last updated by author(s): Feb 20, 2024

Reporting Summary

Nature Portfolio wishes to improve the reproducibility of the work that we publish. This form provides structure for consistency and transparency in reporting. For further information on Nature Portfolio policies, see our Editorial Policies and the Editorial Policy Checklist.

Statistics

For all statistical analyses, confirm that the following items are present in the figure legend, table legend, main text, or Methods section.

n/a	Confirmed	
☐	☒	The exact sample size (*n*) for each experimental group/condition, given as a discrete number and unit of measurement
☐	☒	A statement on whether measurements were taken from distinct samples or whether the same sample was measured repeatedly
☐	☒	The statistical test(s) used AND whether they are one- or two-sided<br>*Only common tests should be described solely by name; describe more complex techniques in the Methods section.*
☒	☐	A description of all covariates tested
☐	☒	A description of any assumptions or corrections, such as tests of normality and adjustment for multiple comparisons
☐	☒	A full description of the statistical parameters including central tendency (e.g. means) or other basic estimates (e.g. regression coefficient) AND variation (e.g. standard deviation) or associated estimates of uncertainty (e.g. confidence intervals)
☐	☒	For null hypothesis testing, the test statistic (e.g. *F*, *t*, *r*) with confidence intervals, effect sizes, degrees of freedom and *P* value noted<br>*Give P values as exact values whenever suitable.*
☒	☐	For Bayesian analysis, information on the choice of priors and Markov chain Monte Carlo settings
☒	☐	For hierarchical and complex designs, identification of the appropriate level for tests and full reporting of outcomes
☒	☐	Estimates of effect sizes (e.g. Cohen's *d*, Pearson's *r*), indicating how they were calculated

Our web collection on statistics for biologists contains articles on many of the points above.

Software and code

Policy information about availability of computer code

Data collection	Primer design involves primer3plus (4.0).<br>Sequencing data pre-processing involves Cell Ranger (5.0.1) and fetch-imgt script, DoubletFinder (2.0.3), Loupe Browser (5.0.1), Loupe VDJ Browser (5.0.0) and a list of web tools (BLAST, UCSC Genome Browser, Blat, Clustal Omega, Expasy Translate).<br>Flow cytometry analysis involves FACSDiva 8.0 Software.
Data analysis	Single cell analysis: SingleR (1.0) , Seurat (4.3.0)<br>Gene length analysis: ggplot2(3.4.2), data.table(1.14.6),viridis(0.6.3),gtools(3.9.2),forcats(0.5.2),dplyr(1.1.2), plyr(1.8.8), cowplot(1.1.1)

For manuscripts utilizing custom algorithms or software that are central to the research but not yet described in published literature, software must be made available to editors and reviewers. We strongly encourage code deposition in a community repository (e.g. GitHub). See the Nature Portfolio guidelines for submitting code & software for further information.

Data

Policy information about availability of data

All manuscripts must include a data availability statement. This statement should provide the following information, where applicable:

- Accession codes, unique identifiers, or web links for publicly available datasets
- A description of any restrictions on data availability
- For clinical datasets or third party data, please ensure that the statement adheres to our policy

All raw scRNAseq and scTCRseq data have been deposited with BioProject: PRJNA742469 and SRA:SRP326193. Custom canine-specific reference files (CanFam3.1, Ensembl v102, see Methods) for use with cellranger count and cellranger vdj are available at http://genomedata.org/10X_canine_ref/.

Research involving human participants, their data, or biological material

Policy information about studies with human participants or human data. See also policy information about sex, gender (identity/presentation), and sexual orientation and race, ethnicity and racism.

Reporting on sex and gender	N/A
Reporting on race, ethnicity, or other socially relevant groupings	N/A
Population characteristics	N/A
Recruitment	N/A
Ethics oversight	N/A

Note that full information on the approval of the study protocol must also be provided in the manuscript.

Field-specific reporting

Please select the one below that is the best fit for your research. If you are not sure, read the appropriate sections before making your selection.

☒ Life sciences ☐ Behavioural & social sciences ☐ Ecological, evolutionary & environmental sciences

For a reference copy of the document with all sections, see nature.com/documents/nr-reporting-summary-flat.pdf

Life sciences study design

All studies must disclose on these points even when the disclosure is negative.

Sample size	Samples size were not predetermined and relied on availability of dog patients in the clinics.
Data exclusions	No collected data was excluded from the analysis.
Replication	N/A. This work reports a protocol, validation and survey of expression of dog TRA/TRB in dog patients. We are not doing a replicable experiment.
Randomization	N/A. This study didn't have experimental groups at the level of samples/organisms/participants
Blinding	N/A. This study didn't have experimental groups, therefore blinding in relation to group allocation is not relevant.

Reporting for specific materials, systems and methods

We require information from authors about some types of materials, experimental systems and methods used in many studies. Here, indicate whether each material, system or method listed is relevant to your study. If you are not sure if a list item applies to your research, read the appropriate section before selecting a response.

Materials & experimental systems

n/a	Involved in the study
☐	☒ Antibodies
☒	☐ Eukaryotic cell lines
☒	☐ Palaeontology and archaeology
☐	☒ Animals and other organisms
☒	☐ Clinical data
☒	☐ Dual use research of concern
☒	☐ Plants

Methods

n/a	Involved in the study
☒	☐ ChIP-seq
☐	☒ Flow cytometry
☒	☐ MRI-based neuroimaging

Antibodies

Antibodies used	List of antibodies clone, isotype, target, supplier,<br>[1] YKIX302.9 , IgG2a k , CD4 , Bio-rad<br>[2] YKIX322.3 , IgG2a k , CD5 , Bio-rad<br>[3] YCATE55.9 , IgG1 k , CD8 , Bio-rad<br>[4] CA2.1D6 , IgG1 , CD21 , Bio-rad<br>[5] P4A10 , IgG1 k , CD25 , eBioscience<br>[6] FJK-16S , IgG2a k , FoxP3 , eBioscience
Validation	[1] Bio-rad website: "Rat anti Dog CD4 antibody...is a monoclonal antibody specific for the canine CD4 cell surface antigen.". Verified by Bio-rad for flow cytometry applications<br>[2] Bio-rad website: "Rat anti Dog CD5 antibody...recognizes canine CD5". Verified by Bio-rad for flow cytometry applications<br>[3] Bio-rad website: "Rat anti Dog CD8 antibody, clone YCATE55.9 was clustered as Canine CD8 in the First Canine Leukocyte Antigen Workshop (Cobbold et al. 1994). YCATE55.9 reacts with a rat cell line transfected with cDNA for canine CD8α (Gorman et al. 1994) and blocks MHC class I dependant T-cell responses in vitro and in vivo." Verified by Bio-rad for flow cytometry applications<br>[4] Bio-rad website: "Mouse anti Canine CD21 antibody, clone CA2.1D6 recognizes canine CD21, also known as Complement receptor type 2. CD21 is a cell surface antigen expressed by canine B lymphocytes". Verified by Bio-rad for flow cytometry applications<br>[5] Thermo website: "This P4A10 monoclonal antibody reacts with canine CD25". "This P4A10 antibody has been pre-titrated and tested by flow cytometric analysis of normal canine peripheral blood cells. "<br>[6] Thermo website: "The FJK-16s antibody reacts with mouse, rat, dog, porcine, bovine and cat Foxp3." 470 flow cytometry publications are listed on the Thermo website

Animals and other research organisms

Policy information about studies involving animals; ARRIVE guidelines recommended for reporting animal research, and Sex and Gender in Research

Laboratory animals	Study was conducted on client-owned companion animals (pet dogs)
Wild animals	N/A
Reporting on sex	Study subjects (dog patients) include individuals from both male and female sex groups.
Field-collected samples	N/A
Ethics oversight	University of Missouri ACUC #30721

Note that full information on the approval of the study protocol must also be provided in the manuscript.

Plants

Seed stocks	N/A
Novel plant genotypes	N/A
Authentication	N/A

Flow Cytometry

Plots

Confirm that:

☐ The axis labels state the marker and fluorochrome used (e.g. CD4-FITC).

☐ The axis scales are clearly visible. Include numbers along axes only for bottom left plot of group (a 'group' is an analysis of identical markers).

☐ All plots are contour plots with outliers or pseudocolor plots.

☐ A numerical value for number of cells or percentage (with statistics) is provided.

Methodology

Sample preparation	Ficoll-separated peripheral blood mononuclear cells (PBMCs) were obtained and cryopreserved. Aspirates were collected from the mandibular lymph nodes and cryopreserved.
Instrument	Beckman Coulter Fortessa X-20 flow cytometer
Software	FACSDiva 8.0 Software
Cell population abundance	Please see "Suppl Table 1. Detailed clinical information for normal canine samples" for cell population distribution.
Gating strategy	Lymphocytes were identified as low FSC and low SSC cells. Subsequent gates were used to define subpopulations of lymphocytes, based on the expression of different surface antigens which were simultaneously stained with different fluorescence-labeled monoclonal antibodies.

☐ Tick this box to confirm that a figure exemplifying the gating strategy is provided in the Supplementary Information.
